# Effects of plant diversity on species-specific herbivory: patterns and mechanisms

**DOI:** 10.1007/s00442-023-05361-6

**Published:** 2023-03-24

**Authors:** M. Bröcher, A. Ebeling, L. Hertzog, C. Roscher, W. Weisser, S. T. Meyer

**Affiliations:** 1 Institute of Ecology and Evolution, University of Jena, Jena, Germany; 2 Thünen Institute of Biodiversity, Brunswick, Germany; 3 Department of Physiological Diversity, Helmholtz Centre for Environmental Research-UFZ, Leipzig, Germany; 4 German Centre for Integrative Biodiversity Research (iDiv) Halle-Jena-Leipzig, Leipzig, Germany; 5 Terrestrial Ecology Research Group, School of Life Sciences, Technical University of Munich, Freising, Germany

**Keywords:** Plant–insect interaction, Ecosystem function, Functional groups, Biodiversity, Grasslands

## Abstract

Invertebrate herbivory can shape plant communities when impacting growth and fitness of some plant species more than other species. Previous studies showed that herbivory varies among plant species and that species-specific herbivory is affected by the diversity of the surrounding plant community. However, mechanisms underlying this variation are still poorly understood. In this study, we investigate how plant traits and plant apparency explain differences in herbivory among plant species and we explore the effect of plant community diversity on these species-specific relationships. We found that species differed in the herbivory they experienced. Forbs were three times more damaged by herbivores than grasses. Variability within grasses was caused by differences in leaf dry matter content (LDMC). Furthermore, higher plant diversity increased herbivory on 15 plant species and decreased herbivory on nine species. Variation within forb and grass species in their response to changing plant diversity was best explained by species’ physical resistance (LDMC, forbs) and biomass (grasses). Overall, our results show that herbivory and diversity effects on herbivory differ among species, and that, depending on the plant functional group, either species-specific traits or apparency are driving those differences. Thus, herbivores might selectively consume palatable forbs or abundant grasses with contrasting consequences for plant community composition in grasslands dominated by either forbs or grasses.

**Supplementary Information:**

The online version contains supplementary material available at 10.1007/s00442-023-05361-6.

## Introduction

Herbivores are omnipresent. While it is well known that mega herbivores can shape entire ecosystems, also relatively low but chronic levels of herbivory by invertebrates can have profound impacts on plants (Bigger and Marvier 1998; Kotanen and Rosenthal 2000). Invertebrate herbivores affect plants directly, by, e.g., altering plant metabolism, triggering leaf abscission, or affecting plant growth and reproduction (Lehndal and Ågren 2015; Zhou et al. 2015; Kozlov and Zvereva 2017). Plants have developed various defense strategies that balance costs and benefits—they either increase resistance (mechanical or chemical) or tolerance (including avoidance) to herbivores (Coley and Barone 1996; Stowe et al. 2000; Rausher 2001; Núñez-Farfán et al. 2007; Agrawal and Weber 2015). To overcome the limitations imposed by plant defenses, herbivores react with different adaptations, including morphological (e.g., specialized mouthparts), behavioral (e.g., trenching), and physiological responses (e.g., excretion, sequestration, and detoxification; Ehrlich and Raven 1964; Karban and Agrawal 2002; Hopkins et al. 2009; Pentzold et al. 2014). The set of strategies used by a plant species and its associated herbivores are highly species-specific, likely leading to high variability in herbivory among plant species (Cárdenas et al. 2014; Turcotte et al. 2014).

Species-specific herbivory strongly impacts entire plant communities. Plant species differ in their attractiveness to herbivores, e.g., due to the nutritional quality, defense, or apparency (plant height, color, odor, abundance, and biomass; Scherber et al. 2010; Carmona et al. 2011; Loranger et al. 2012, 2013). Consequently, plant species differ in the loss of plant biomass to herbivores, altering the outcome of plant–plant competition (Huntly 1991; Wolf et al. 2008; Stein et al. 2010). Fast-growing, poorly defended species might be heavily consumed by herbivores, allowing other, slower-growing but better-defended species to persist, thereby affecting plant community composition and diversity (Coley et al. 1985). Plant diversity can be stabilized if dominant plant species are under stronger herbivory pressure than subordinate plant species (Huntly 1991; Lamarre et al. 2012; Castagneyrol et al. 2014; Koerner et al. 2018).

Herbivory alters plant community properties, but also plant community properties shape plant–herbivore interactions. Community properties can alter a plant’s susceptibility to herbivores directly and indirectly via modifying the consumer communities. Direct effects occur, for example, if a higher density of a plant species either increases or decreases herbivory damage. Negative density-dependence is possible if the population of a specialized herbivore remains constant despite increasing host–plant quantities. Thus, fewer herbivores per plant individual accumulate (Resource Dilution Hypothesis; Otway et al. 2005). Alternatively, a higher density of the plant species may make it an easier target to be found that is more heavily consumed (Resource Concentration Hypothesis; Root 1973). Moreover, the composition and diversity of a plant community can further modify the herbivory on a given plant species (Underwood et al. 2014). So-called associational effects can be negative, for example, if the neighboring plant species are more attractive to herbivores than the respective target species (associational resistance) or, positively, if the neighboring plants are less attractive than the target species (associational susceptibility). Consequently, plant community composition modifies the herbivory pressure on a target plant and can thus increase or decrease species-specific differences.

Indirect effects of plant community properties occur when a plant’s herbivory is modified by changing abundance and species richness of consumers. For example, productivity can promote herbivory by providing more resources that can support higher herbivore abundances (More Individuals Hypothesis; Srivastava and Lawton 1998; Borer et al. 2012). Similarly, resource diversity can enhance herbivory by accumulating specialized herbivores on different resources (Resource Specialization Hypothesis; Hutchinson 1959; Hurlbert 2004). Moreover, the structural complexity of plant communities modifies the microclimate and the availability of microhabitats, thus impacting the behavior of both herbivores and predators. Especially, predators are thought to profit from the additional refuges and alternative resources, leading to a greater diversity and abundance, thus increasing top–down control on herbivores and reducing herbivory rates (Enemies Hypothesis; Russell 1989). Altogether, the traits of each plant species, the abundance distribution within a plant community, associational effects, plant productivity, resource diversity, and structural complexity are affected by plant diversity. Consequently, plant diversity is a crucial factor for predicting species-specific herbivory. Higher plant diversity increases productivity (Hector et al. 1999; Roscher et al. 2005), structural complexity, and complementarity between plant species (Marquard et al. 2009; Zuppinger-Dingley et al. 2014) while decreasing the availability of individual plant species. Simultaneously, plants respond to increasing plant diversity by, e.g., adjusting plant height (shoot length), shoot biomass, specific leaf area (SLA), nitrogen concentrations, or phenology (Gubsch et al. 2011; Roscher et al. 2011).

Because of the complex changes that plant diversity imposes at the level of plant communities and individual plant species, it remains challenging to predict how species-specific herbivory responds to the diversity of the surrounding plant community. Previous studies found no consistent effects but highlighted the importance of species identity effects (Scherber et al. 2006; Schuldt et al. 2010; Vockenhuber et al. 2013; Loranger et al. 2014; Hahn et al. 2017; Fricke et al. 2022). Especially, studies exploring herbivory at the species level often struggle with small data sets, short time periods, biases in the selection of plant species, and young or transient plant communities (Kozlov and Zvereva 2017; Meyer et al. 2017). Consequently, it remains unclear how herbivory on individual plant species changes in communities of differing plant diversity and which mechanisms cause the plant diversity effects.

Here, we use data from a large-scale grassland biodiversity experiment from multiple years and seasons to (1) understand general differences in species-specific levels of herbivory, (2) test the effect of plant diversity on species-specific herbivory, and (3) explore the mechanisms underlying the species-specific response to changing plant diversity. We ask the following questions:

1. Do plant species differ in their level of herbivory and what are the underlying mechanisms? We expect that species differ in their overall level of herbivory because of differences in plant traits and their apparency in a community.
2. Is species-specific herbivory affected by plant diversity? We expect that species-specific herbivory changes with plant diversity due to variation among plant species in nutritional traits, defense traits, or their apparency in a plant community.
3. What are the mechanisms underlying the plant diversity–species-specific herbivory relationship? Plant traits, species densities, associational effects, resource diversity, and structural complexity change with plant diversity and could facilitate or mitigate plant diversity effects on herbivory.

## Methods

### Field site

The study was conducted in the framework of “The Jena-Experiment” (Thuringia, Germany, 50°55´ N, 11°35´ E; 130 m a. s. l.), one of the largest long-term biodiversity experiments worldwide that was established in 2002 on former arable land (Roscher et al. 2004). Here, we used the trait-based experiment (TBE) established in 2010 on the same field site (see Ebeling et al. 2014 for a detailed description of the TBE). Twenty non-leguminous plant species (7 grasses and 13 forbs) were selected from a larger species pool and sown on 138 plots (3.5 m × 3.5 m) of different plant species richness (PSR) (1, 2, 3, 4, and 8 species per plot) and varying species compositions. Plant species were selected based on a principal component analysis, and three partially overlapping species pools were defined, each containing eight species (Table 1). Plant species span a gradient in spatial and temporal resource acquisition traits. Pool A represents species with different spatial resource use niches. Pool B represents species with different temporal resource use niches. Finally, pool C represents species that represent the extremes in spatial and temporal resource use niches within the species pool. Each plot only contained species from a single pool; thus, the three pools represent independent replicates. The plant communities were mown twice a year in early June and September and weeded three times per year in spring, summer, and autumn to maintain the biodiversity gradient. Plots were arranged in three blocks.

**Table 1:** List of all plant species and their average level (mean) of percentage herbivory (%) and consumed biomass (g m^−2^) with their respective standard deviations across all years, seasons, and plots, sorted by their corresponding species pool

Pool	Species acronyms	Species	Functional group	Percentage herbivory	*σ*	Consumed biomass	*σ*	*n*
A	Ave Pub	*Avenula pubescens*	Grass	0.81	1.37	0.14	0.30	143
	Cen Jac	*Centaurea jacea*	Forb	1.39	1.57	0.42	0.64	128
	Fes Rub	*Festuca rubra*	Grass	0.59	1.26	0.11	0.32	121
	Kna Arv	*Knautia arvensis*	Forb	0.67	0.79	0.52	1.02	149
	Leu Vul	*Leucanthemum vulgare*	Forb	1.10	1.81	0.25	0.75	141
	Phl Pra	*Phleum pratense*	Grass	0.45	0.66	0.34	0.79	140
	Pla Lan	*Plantago lanceolata*	Forb	2.90	2.83	1.02	1.04	143
	Poa Pra	*Poa pratensis*	Grass	0.40	0.68	0.04	0.13	117
B	Ant Odo	*Anthoxanthum odoratum*	Grass	0.72	1.25	0.07	0.20	104
	Dac Glo	*Dactylis glomerata*	Grass	0.42	0.58	0.23	0.39	156
	Ger Pra	*Geranium pratense*	Forb	1.33	1.61	0.47	0.70	135
	Hol Lan	*Holcus lanatus*	Grass	0.23	0.41	0.06	0.13	134
	Leu Vul	*Leucanthemum vulgare*	Forb	0.85	1.37	0.22	0.48	150
	Phl Pra	*Phleum pratense*	Grass	0.46	0.74	0.27	0.55	135
	Pla Lan	*Plantago lanceolata*	Forb	3.37	3.10	0.84	0.79	138
	Ran Acr	*Ranunculus acris*	Forb	0.92	1.66	0.16	0.23	133
C	Ant Odo	*Anthoxanthum odoratum*	Grass	0.50	0.62	0.08	0.14	133
	Ant Syl	*Anthriscus sylvestris*	Forb	0.30	0.96	0.03	0.11	110
	Cir Ole	*Cirsium olearaceum*	Forb	1.56	1.82	1.24	1.81	115
	Gle Hed	*Glechoma hederacea*	Forb	0.79	1.02	0.02	0.03	120
	Pru Vul	*Prunella vulgaris*	Forb	1.71	2.15	0.89	1.40	91
	Rum Ace	*Rumex acetosa*	Forb	5.29	5.05	0.20	0.32	144
	San Off	*Sanguisorba officinalis*	Forb	0.59	0.82	0.10	0.18	79
	Ver Cha	*Veronica chamaedrys*	Forb	0.53	0.78	0.31	1.13	133

### Herbivory measurements

We measured herbivory by invertebrates and small mammals (large herbivores were excluded by a fence from the field site) between 2012 and 2016 twice per year during the peak biomass production end of May and end of August. For the herbivory assessment, we used plant material from a randomly taken biomass sample: Within an area of 20 × 50 cm, we cut the vegetation at 3 cm above the ground and sorted samples by species. We randomly drew a maximum of 30 leaves from the sorted biomass samples for each plot × species combination. We investigated each leaf using a magnifying glass and estimated herbivory by comparing to template cards with shapes of known surface area. We estimated herbivory in mm^2^ as a total value of four damage types: chewing damage (1), rasping damage (2), sap-sucking damage (3), and leaf-mining damage (4). In 2016, all damage types were estimated separately, and between 2012 and 2015, only a total value of herbivory including all four types was recorded. Finally, we measured leaf area with a leaf area meter (LI-3000C Area Meter, LI-COR Biosciences, Lincoln, Nebraska, USA). Since the area meter measures only the leftover area, which includes rasping, sap-sucking and mining, but not chewing damage, we estimated the original leaf area by adding the area lost to chewing damage (for 2016). Because we recorded chewing damage only in 2016, we needed to use a plant species-specific correction factor to estimate the proportion of chewing damage between 2012 and 2015. We obtained the correction factors from the chewing damage measured in monocultures of the field site (Loranger et al. 2014).

We calculated the percentage herbivory by dividing the damaged leaf area by the measured leaf area after correcting for chewing damage and multiplied by 100. To calculate the consumed biomass in gram dry weight, we multiplied the leaf biomass (see “Measures of plant performance”) for each species with the respective proportional herbivory.

### Measures of plant performance

After measuring herbivory, we dried all samples at 70 °C for 48 h and weighed them at the species level for both seasons and all years. We converted the total biomass of each species into the respective leaf biomass (for simplicity, hereafter called biomass or plant biomass) by multiplying total biomass with a conversion factor C. The conversion factor C is the quotient of the species-specific leaf area ratio (LAR) and the specific leaf area (SLA) obtained from the monocultures (data collected between 2002 and 2009, average values over repeated measurements have been calculated for each season and species). In addition to biomass, we visually estimated plant cover twice a year during peak biomass using a modified Londo scale (Londo 1976) on the whole-plot area excluding the outer 20 cm of the plot margin. Numerical values for species cover were coded as 0.5 (< 1%), 3 (1–5%) 10 (6–15%), 20 (16–25%), 30 (26–35%), 40 (36–45%), 50 (46–55%), 60 (56–65%), 70 (66–75%), 80 (76–85%), and 90 (> 85%).

### Plant traits

We measured leaf traits for all species twice in 2012 at peak standing biomass (late May and late August), which is consistent with the time of our herbivory measurements. From each species x plot combination, we sampled 5–10 young, fully expanded leaves and stored them in moistened tissue in sealed plastic bags at 4 °C overnight for rehydration. After removing any water droplets with dry tissue, we weighed the fresh weight of the leaves, followed by drying the leaves at 70 °C for 48 h and weighing them again. Leaf dry matter content (LDMC; mg g^−1^) was calculated as the ratio of dry weight to fresh weight. Finally, we ground leaf samples with a mixer mill (MM200, Retsch, Germany) and analyzed samples with an elemental analyzer (FlashEA 112, Thermo Electron, Italy) to obtain the leaf nitrogen concentration per mass (N_leaf_; mg N g_dw_^−1^).

### Statistical analysis

Statistical analyses were performed in R version 4.0.3 (R Core Team 2021). We used linear models and linear mixed-effects models (Type I Sums of Squares; lme4 package, lmerTest; Bates et al. 2015; Kuznetsova et al. 2017) with either percentage herbivory, consumed biomass or the change in percentage herbivory, or consumed biomass with PSR, as response variables. We log-transformed percentage herbivory and consumed biomass (after adding constants of 0.001 and 0.0001, respectively) to improve the normality of residuals. To show the response of herbivory to plant apparency and traits with appropriate standard errors for each functional group and to test if these differ significantly from zero, we extracted the functional group means and functional group slopes by removing the main effects intercept and the main effect of plant apparency and traits (formula: herbivory ~ − 1 + FG + FG:plant parameter; following Schielzeth 2010). Variance compounds of random terms can be found in Table S1.

### Do species differ in their level of herbivory and what are the underlying mechanisms?

To test for differences in the average level of herbivory damage experienced by a plant species and the underlying mechanisms (Question 1), we used linear mixed-effects models with either percentage herbivory or consumed biomass as response. We fitted eight models (four for percentage herbivory; four for consumed biomass) containing either biomass (log-transformed, after adding a constant of 1), cover, N-concentration or LDMC and their interaction with functional group identity as explanatory variable. To account for spatial and temporal non-independence of the data, we used plot and sampling times (combination between sampling year and season) in addition to species identity as random terms (formula: herbivory ~ parameter*FG + (1|plot) + (1|sampling time) + (1|species), Table S1).

### Is species-specific herbivory affected by plant diversity?

To test if species-specific herbivory responses to changing plant diversity (Question 2), we used percentage herbivory or consumed biomass as response variables and tested the effect of PSR (log2-transformed), species identity, year, and season. The explanatory variables were fitted in this order along with all possible interactions after the main effects. To account for spatial non-independence of the data, we used plot nested in block as random term. We analyzed the three species pools separately (herbivory ~ PSR*species*year*season + (1|block/plot)).

### What are the mechanisms underlying the plant diversity–herbivory relationship?

To test if the change in species-specific herbivory with plant diversity depends on their traits or apparency (Question 3), we calculated the response of percentage herbivory and consumed biomass to PSR for each plant species (slope of herbivory over PSR). We used linear mixed-effects models to assess the effect of biomass, cover, N_leaf_, and LDMC and their interaction with functional group identity on these slopes. To account for temporal non-independence of the data, we used sampling time as a random term (PSR slope herbivory ~ parameter*FG + (1|sampling time)).

Finally, to test whether the plant diversity induced changes in species-specific herbivory and plant apparency or traits correlate, we fitted a series of models. First, to extract the response of herbivory to changing PSR (slope of herbivory over PSR), we used PSR, species identity, year, and season as explanatory variables. The explanatory variables were fitted in this order along with all possible interactions. To account for spatial non-independence of the data, we used plot nested in block as random term. Second, we extracted the response of biomass, cover, N-concentration, and LDMC to changing PSR (slope of apparency/trait over PSR), using the same model structure. Third, to assess the correlation between both the slopes over herbivory and the slopes of the vegetation-related explanatory variables, we used the slope of apparency and traits over PSR and their interaction with functional group as explanatory variables (PSR slope herbivory ~ PSR slope parameter*FG).

## Results

### Do species differ in their average level of herbivory and what are the underlying mechanisms?

Species differed strongly in the average herbivory they experienced across all years, seasons, and plots. A large range was apparent both in percentage herbivory (max. 5.29% *Rumex acetosa,* min*.* 0.23% *Holcus lanatus*; Table 1) and consumed biomass (max. 1.24 g m^−2^ *Cirsium olearaceum,* min. 0.02 g m^−2^ *Glechoma hederacea*). Forb species showed an average percentage herbivory of 1.62% and consumed biomass of 0.45 g m^−2^, which was about three-times higher herbivory than in grasses.

**Fig. 1:**
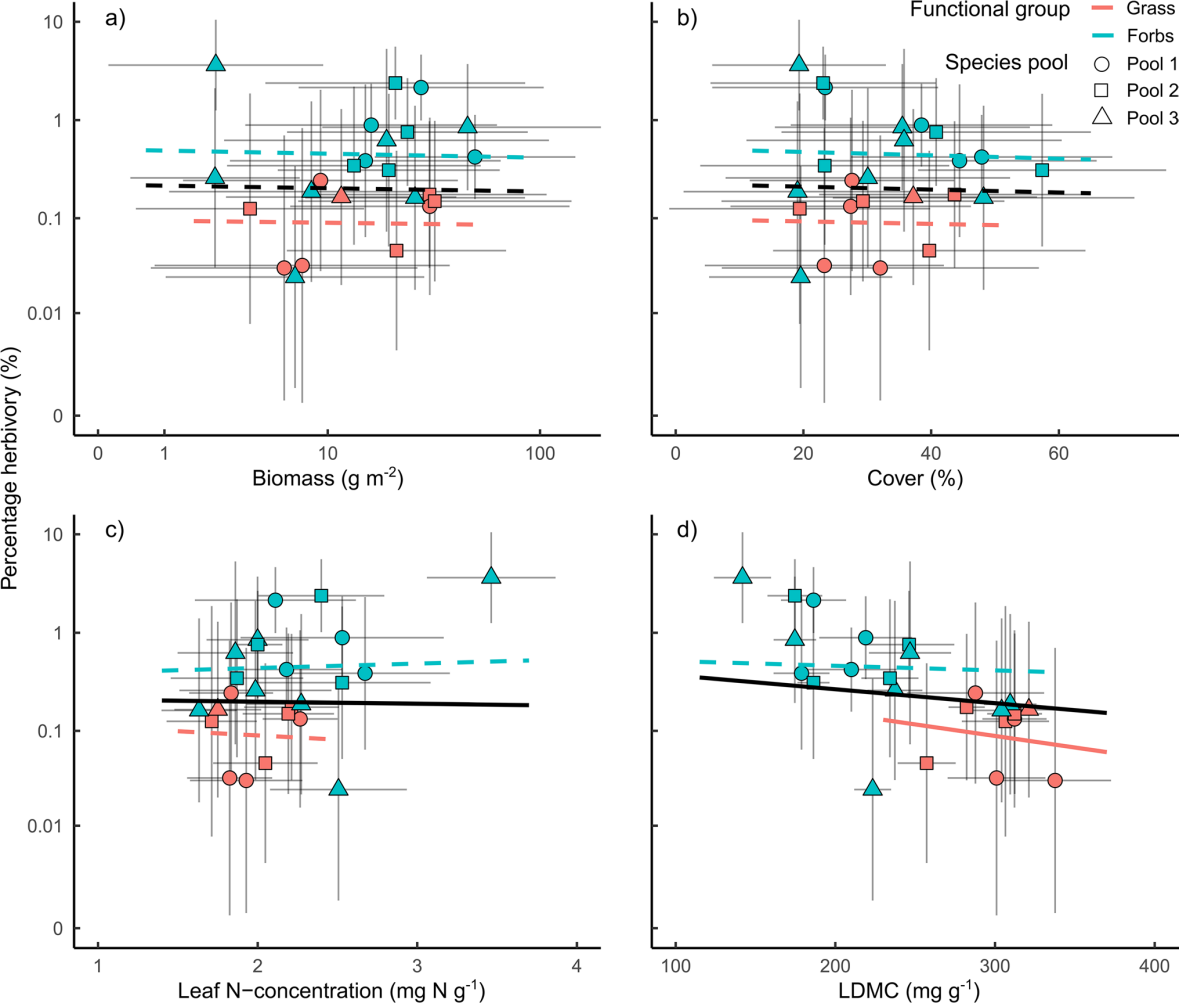
Effect of **a** biomass, **b** cover, **c** leaf N-concentration, and **d** LDMC on percentage herbivory. Each point represents one species x pool combination. The species pool is indicated by the shape of the points (A = circle, B = triangle, and C = square). Lines are predictions from the models and indicate significance (solid lines: *P* < 0.05; dashed lines: *P* > 0.05, Table 2). Response of herbivory for the average of both plant functional groups is shown in black, for forbs in blue, and for grasses in red. Vertical and horizontal grey lines indicate the standard deviation per species. X-axis of the upper left figure and all Y-axes are logarithmic

Among the tested plant properties, only LDMC explained parts of the intra-specific variation in percentage herbivory (Fig. 1, Table 2). Plant species with low LDMC showed significantly higher levels of herbivory, explaining 12% of variation in percentage herbivory (Fig. 1, Table 2, Figure S1, Table S2). Specifically, percentage herbivory decreased with higher LDMC for grasses, while for forbs, the effects of LDMC were less pronounced (Fig. 1). In contrast, variation among species in the amount of consumed biomass was best explained plant species biomass (*R*^2^ = 0.40) and plant cover (*R*^2^ = 0.16, Figure S1, Table S2). Investigating the individual functional groups revealed that consumed biomass increased with plant species biomass and cover in grasses and forbs, while in grasses consumed biomass increased also with higher LDMC.

**Table 2:** Summary statistics for linear mixed-effects models testing the effect of biomass, cover, leaf N-concentration, and LDMC and their interaction with functional group identity (FG) on percentage herbivory (Fig. 1) and the change in percentage herbivory with plant species richness (PSR) (Fig. 4)

Explanatory variable	Percentage herbivory	PSR slope percentage herbivory
	*F* value	*F* value
Biomass	*F*_1.2510_ = 0.6	*F*_1.237_ = 3.8*
FG	*F*_1.20_ = 10.2**	*F*_1.230_ = 0.0
FG:biomass	*F*_1.3017_ = 0.1	*F*_1.240_ = 2.9
cover	*F*_1.823_ = 0.6	*F*_1.240_ = 1.3
FG	*F*_1.20_ = 10.3**	*F*_1.230_ = 0.0
FG:cover	*F*_1.2222_ = 0.1	*F*_1.237_ = 0.8
N-concentration	*F*_1.46_ = 8.9**	*F*_1.231_ = 2.0
FG	*F*_1.20_ = 10.2**	*F*_1.230_ = 0.2
FG:N-concentration	*F*_1.2297_ = 1.9	*F*_1.231_ = 0.1
LDMC	*F*_1.23_ = 12.3**	*F*_1.231_ = 2.9
FG	*F*_1.21_ = 8.1**	*F*_1.231_ = 2.7
FG:LDMC	*F*_1.1958_ = 2.1	*F*_1.238_ = 3.7

For each plant parameter, we fitted a separate model

Asterisks indicate significance: *P < 0.05, **P < 0.01, ***P < 0.001

### Is species-specific herbivory affected by plant diversity?

**Fig. 2:**
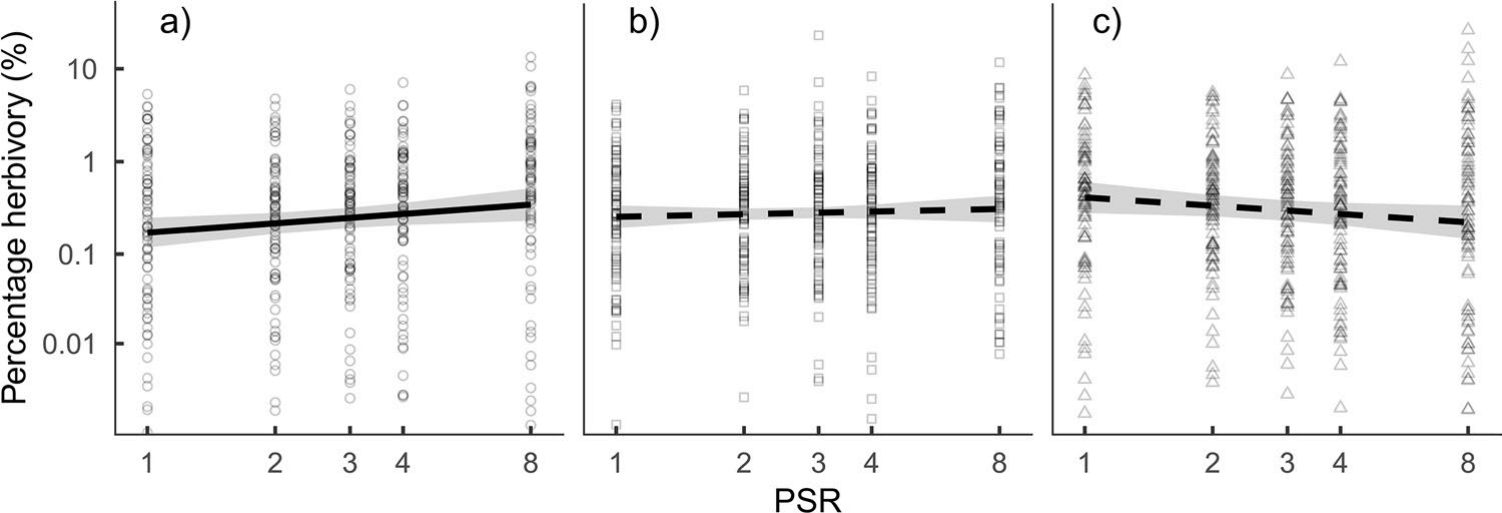
Effect of plant species richness on percentage herbivory for three different species pools. Lines are predictions from the models and indicate significance (solid lines: *P* < 0.05; dashed lines: *P* > 0.05, Table 3). Bands indicate 95% confidence intervals. Axes are logarithmic

**Fig. 3:**
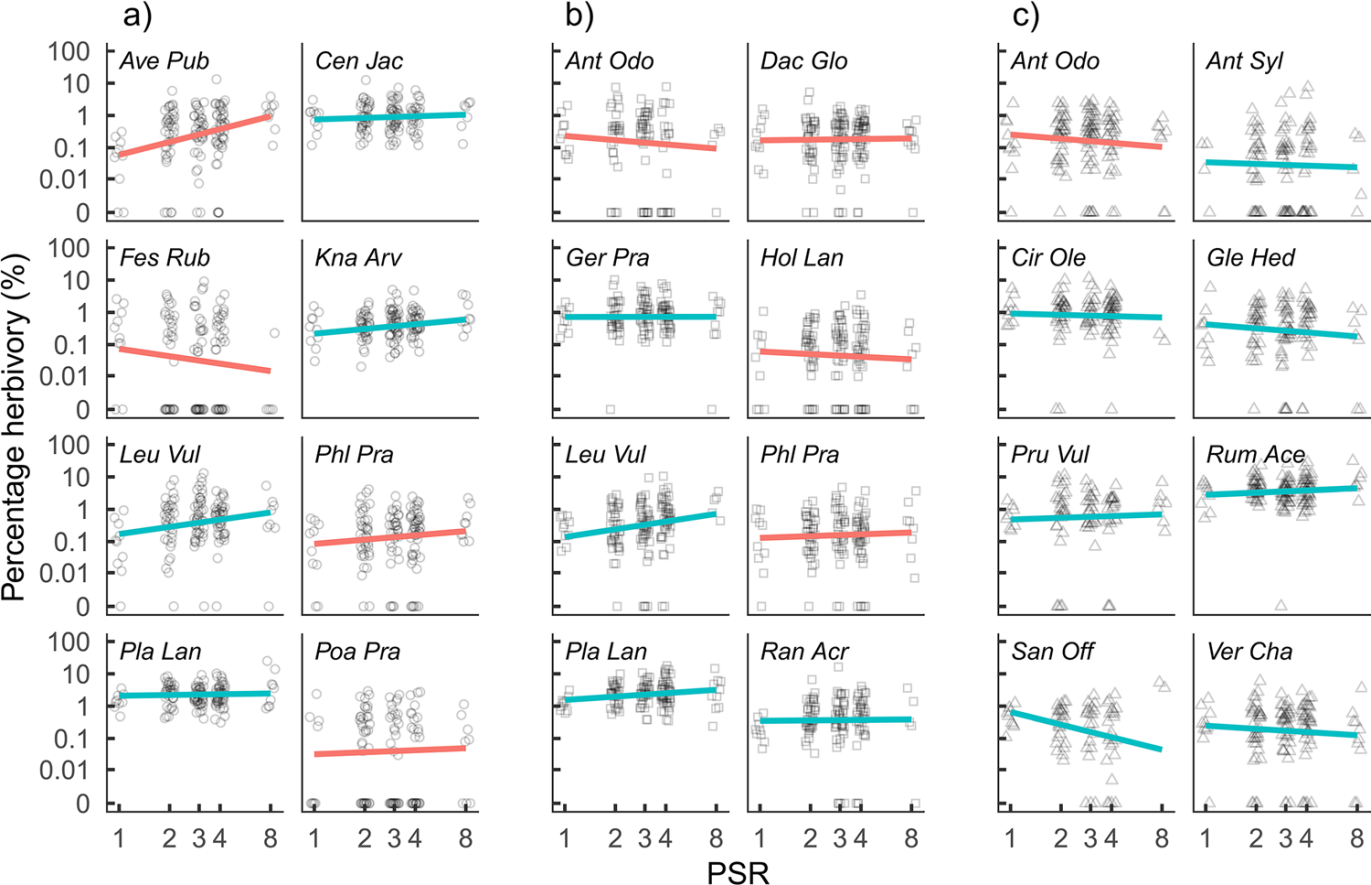
Effect of plant species richness on percentage herbivory for all species x pool combinations. Lines are predictions from the models and the color of the lines indicate the functional group with forbs shown in blue and grasses in red. Axes are logarithmic

There was high variability in herbivory within plant species (Table 1) that could be partially related to effects of PSR on herbivory. Effects of PSR on percentage herbivory varied between species and species pools. In pool A and B, percentage herbivory increased on average from 0.17 and 0.25% in monocultures to 0.34 and 0.40% in 8 species mixtures, respectively, while it decreased on average from 0.41 to 0.22% in pool C (Fig. 2). However, effects in pool B and C were not statistically significant (Table 3). Furthermore, there were strong differences among species in their response to changing PSR (significant interaction between PSR and species identity in pool A and B, Table 3) ranging from species that suffered 6 times more herbivory in mixture than monoculture (change from 0.15 to 0.95%, *Avenula pubescens*, Fig. 3) to species that suffered 6 times less (from 0.27 to 0.04%, *Sanguisorba officinalis*). Overall, grass and forb species changed similarly with PSR; percentage herbivory increased in 10 out of 15 forb species and four out of nine grasses with higher PSR (Figure S4). In addition, percentage herbivory differed between years (pool A and B) and seasons (pool B and C), and there was variability in effects of PSR on percentage herbivory between seasons, years, and species (see higher order interactions between PSR, species, season, and year in Table 3).

**Table 3:** Summary statistics for linear mixed-effects models testing the effect of plant species richness (PSR), species identity, year, season, and all interactions on percentage herbivory for the three different species pools separately

	Percentage herbivory		
	Pool A	Pool B	Pool C
Explanatory variable	*F* value	*F* value	*F* value
PSR	*F*_1.38_ = 8.4**	*F*_1.44_ = 1.4	*F*_1.48_ = 1.4
species	*F*_7.537_ = 88.4***	*F*_7.585_ = 76.7***	*F*_7.499_ = 90.8***
year	*F*_4.1037_ = 3.7**	*F*_4.1045_ = 5.0***	*F*_4.893_ = 2.1
season	*F*_1.1038_ = 0.0	*F*_1.1045_ = 19.8***	*F*_1.894_ = 12.6***
PSR:species	*F*_7.413_ = 3.8***	*F*_7.463_ = 2.1*	*F*_7.419_ = 0.9
PSR:year	*F*_4.1037_ = 0.6	*F*_4.1046_ = 1.2	*F*_4.894_ = 2.1
species:year	*F*_28.1036_ = 2.9***	*F*_28.1045_ = 2.9***	*F*_28.894_ = 3.7***
PSR:season	*F*_1.1040_ = 3.7	*F*_1.1048_ = 6.2*	*F*_1.893_ = 2.6
species:season	*F*_7.1038_ = 3.4***	*F*_7.1044_ = 6.3***	*F*_7.892_ = 10.9***
year:season	*F*_4.1036_ = 22.7***	*F*_4.1044_ = 22.9***	*F*_4.893_ = 18.1***
PSR:species:year	*F*_28.1036_ = 2.0***	*F*_28.1046_ = 1.2	*F*_28.894_ = 1.2
PSR:species:season	*F*_7.1041_ = 1.8	*F*_7.1046_ = 1.1	*F*_7.894_ = 2.7**
PSR:year:season	*F*_4.1039_ = 2.6*	*F*_4.1045_ = 0.4	*F*_4.894_ = 0.5
species:year:season	*F*_28.1037_ = 4.7***	*F*_28.1046_ = 2.0**	*F*_28.896_ = 2.4***
PSR:species:year:season	*F*_28.1038_ = 1.7*	*F*_28.1047_ = 1.6*	*F*_28.897_ = 1 .0

Asterisks indicate significance: *P < 0.05, **P < 0.01, ***P < 0.001

Consumed biomass significantly decreased with higher plant species richness across all species, seasons, years, and in all three pools (Figure S2, Table S3). Grasses and forbs showed similar changes in consumed biomass with increasing PSR (Figure S3, Figure S4). While plant species differed significantly in their response of consumed biomass to increasing PSR (PSR:species interaction, Table S3), 23 out of 24 species showed a decrease of consumed biomass with higher PSR.

### What are the mechanisms underlying the plant diversity–herbivory relationship?

**Fig. 4:**
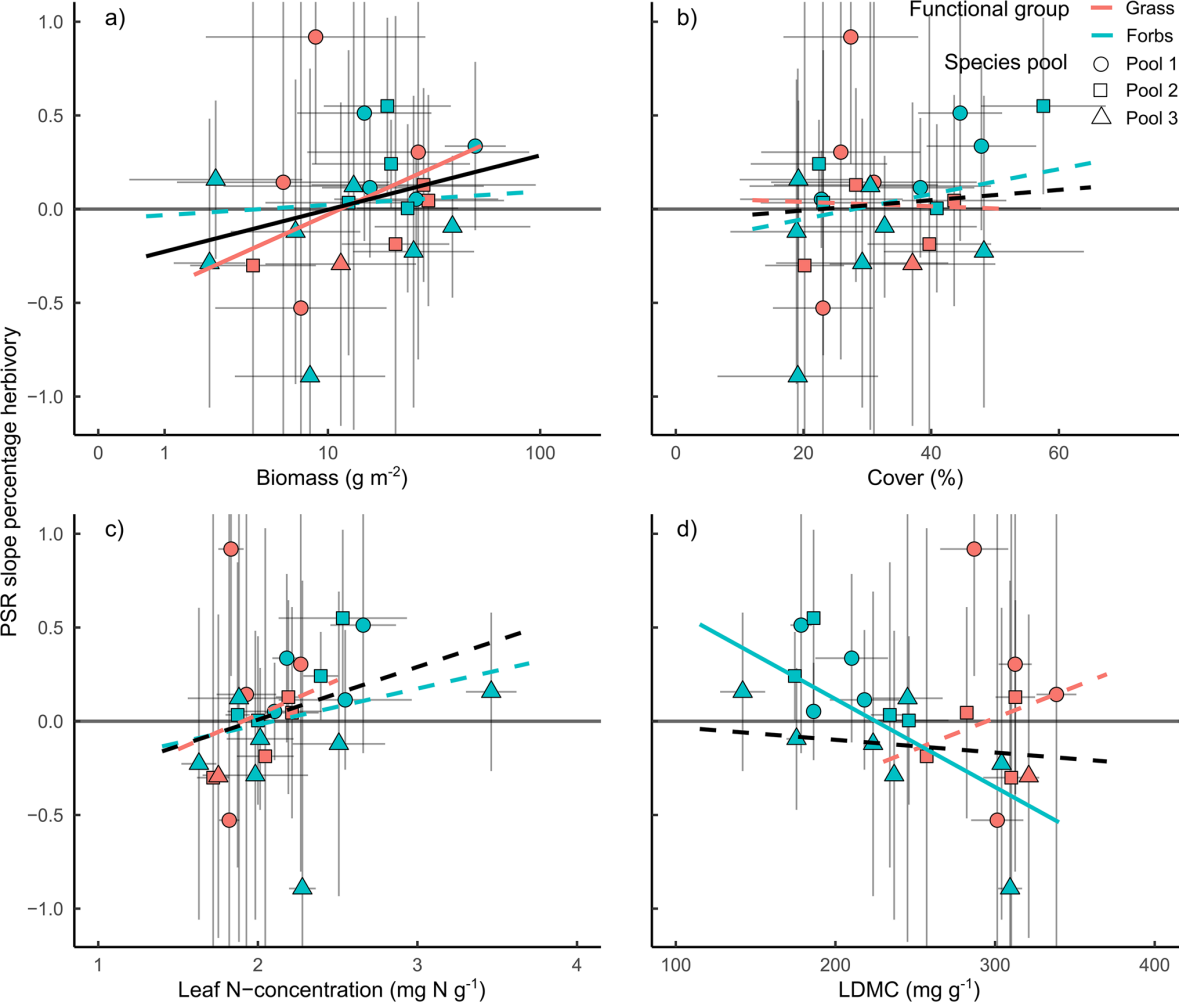
Effect of **a** biomass, **b** cover, **c** leaf N-concentration, and **d** LDMC on the change in percentage herbivory with plant species richness (PSR). Each point represents one species x pool combination. The species pool is indicated by the shape of the points (A = circle, B = triangle, C = square). Lines are predictions from the models and indicate significance (solid lines: *P* < 0.05; dashed lines: *P* > 0.05, Table 2). Response of herbivory for the average of both plant functional groups are shown in black, for forbs in blue, and for grasses in red. Vertical and horizontal grey lines indicate the standard deviation per species. X-axis of the upper left figure and all Y-axes are logarithmic

Variation among species in PSR effects on percentage herbivory could be explained by species biomass, but not species cover, N-concentration, or LDMC. Specifically, plant species with an average high biomass doubled herbivory in mixture compared to monoculture (from 0.49 to 1.28%, *Knautia arvensis,* average biomass of 50.3 g m^−2^), while species with low biomass halved herbivory (from 0.96 to 0.46%, *Glechoma hederacea,* average biomass of 2.2 g m^−2^, Fig. 4). In contrast, variation among species in PSR effects on consumed biomass could not be explained by any of the variables (Table S2). Considering the functional groups separately showed that the biomass effect on the PSR slope in percentage herbivory and consumed biomass was stronger for grasses (significantly different from zero) than for forbs. In addition, forb species with a low LDMC experienced more damage in high compared to low diverse plant communities (Fig. 4, Figure S5). In contrast, grass species with high LDMC suffered from an increase in PSR—however, this effect was neither significant for percentage herbivory nor for consumed biomass (significant interaction between FG and LDMC, Table 2, Table S2).

**Fig. 5:**
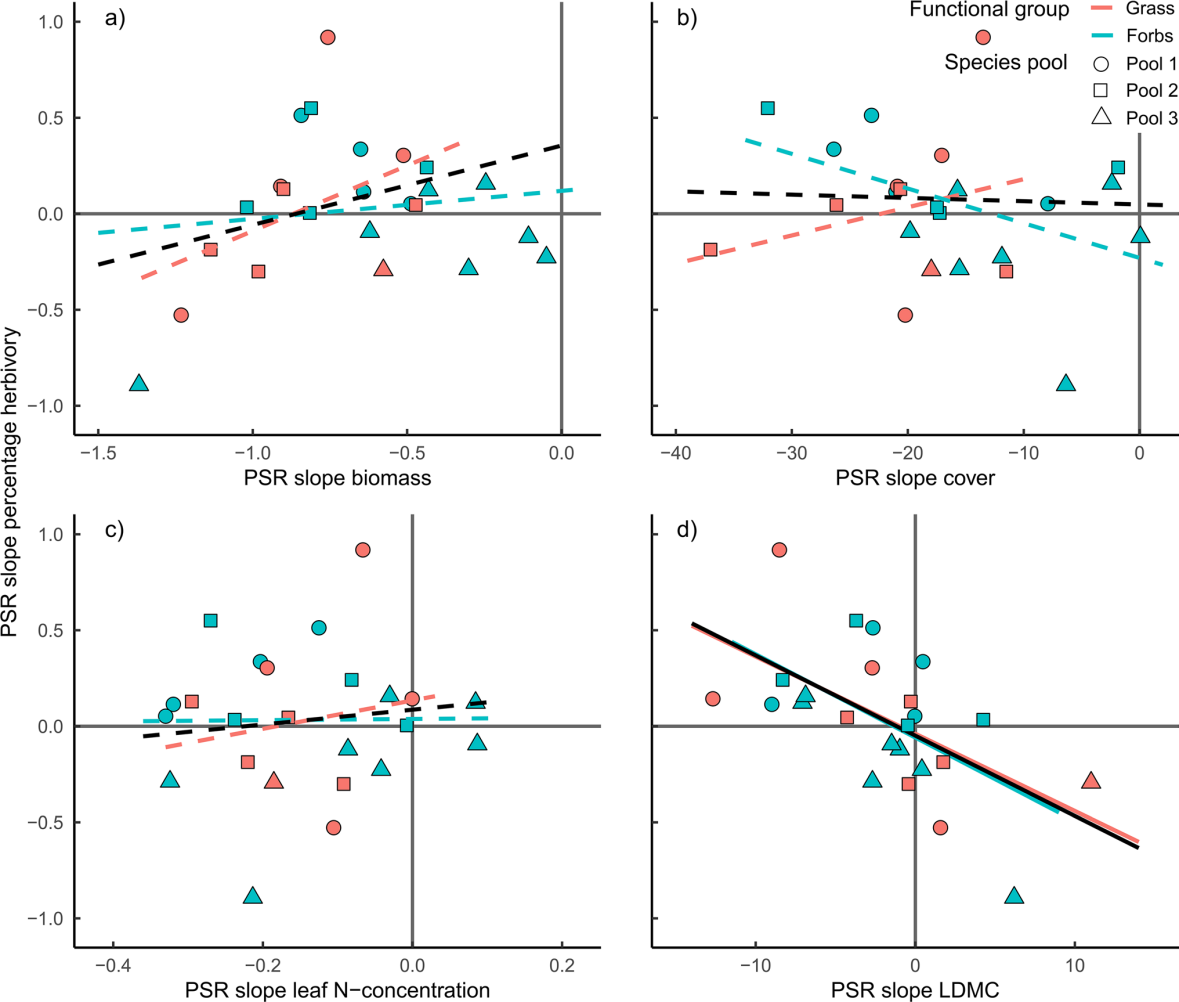
Effect of species-specific change in **a** biomass, **b** cover, **c** leaf N-concentration, and **d** LDMC with plant species richness (PSR) on the change in percentage herbivory with PSR. Each point represents one species x pool combination. The species pool is indicated by the shape of the points (A = circle, B = triangle, and C = square). Lines are predictions from the models and indicate significance (solid lines: *P* < 0.05; dashed lines: *P* > 0.05, Table 4). Response of herbivory for the average of both plant functional groups is shown in black, for forbs in blue, and for grasses in red

Average plant-related variables could partially explain average herbivory and changes of herbivory with PSR. While all plant variables changed with PSR, only PSR induced changes in LDMC could further explain how herbivory of a species changed with PSR. Plant species that increased their LDMC with increasing PSR experienced less damage by herbivores in high compared to low diverse plant mixtures, and the other way around. The pattern was strong and robust for both measures, percentage herbivory (*P* = 0.0043, *R*^2^ = 0.31), and consumed biomass (*P* = 0.0079, *R*^2^ = 0.29, Fig. 5, Table 4, Figure S6, Table S4). The decrease in consumed biomass with increasing PSR was in addition related to the decrease in biomass with increasing PSR. All effects of changing plant parameters with PSR were consistent in grasses and forbs (Fig. 5, Figure S6).

**Table 4:** Summary statistics for linear models testing the effect of the change in percentage herbivory with plant species richness [slope of percentage herbivory and plant species richness (PSR)] on the change in biomass, cover, leaf N-concentration, and LDMC with PSR (slope of biomass/cover/N-concentration/LDMC and PSR) and their interactions with functional group identity (FG)

Explanatory variable	PSR slope percentage herbivory
	*F* value
PSR slope biomass	*F*_1.20_ = 1.2
FG	*F*_1.20_ = 0.1
FG:PSR slope biomass	*F*_1.20_ = 0.9
PSR slope cover	*F*_1.20_ = 1.0
FG	*F*_1.20_ = 0.2
FG:PSR slope cover	*F*_1.20_ = 2.7
PSR slope N-concentration	*F*_1.20_ = 0.0
FG	*F*_1.20_ = 0.0
FG:PSR slope N-concentration	*F*_1.20_ = 0.1
PSR slope LDMC	*F*_1.20_ = 10.3**
FG	*F*_1.20_ = 0.0
FG:PSR slope LDMC	*F*_1.20_ = 0.0

Asterisks indicate significance: *P < 0.05, **P < 0.01, ***P < 0.001

## Discussion

Previous studies showed variation in herbivory and mixed results for diversity effects on herbivory (Scherber et al. 2006; Schuldt et al. 2010; Vockenhuber et al. 2013; Loranger et al. 2014; Hahn et al. 2017; Fricke et al. 2022). Here, we studied species-specific herbivory in one of the largest grassland biodiversity experiments (Jena Experiment) and found that the average herbivory strongly differed among plant species and functional groups (forbs species were more damaged by herbivores than grasses). These differences were explained by plant traits and plant apparency in the communities. In detail, plant species with, on average, low LDMC were damaged most by herbivores and these effects were stronger for grasses than forbs. Furthermore, effects of PSR on herbivory differed among plant species, ranging from negative (*n* = 9) to positive (*n* = 15). In particular, plant species with high average biomass suffered from increasing PSR (increase in herbivory) and that effect was stronger for grasses than forbs. Forbs showed a decrease in herbivory with increasing PSR if they had high LDMC. In addition to the effect of average LDMC, also the change in LDMC with PSR explained how herbivory changed with PSR. This was observed for both forbs and grasses.

### Plant traits and apparency affect species-specific herbivory

We found that plant species differed strongly in the herbivory damage they experienced as did previous studies (e.g., Marquis et al. 2001; Kozlov et al. 2015; Těšitel et al. 2021). Regarding the underlying mechanisms, species with low LDMC were damaged the most, caused by their lower physical resistance (Coley and Barone 1996; Poorter et al. 2009; Schuldt et al. 2012). Similar effects of LDMC or similar defensive traits (e.g., leaf toughness, lignin concentration) on herbivory are frequently observed in grasslands (Loranger et al. 2012; Těšitel et al. 2021). In addition to LDMC, variability among plant species herbivory was also explained by plant apparency. Plant species with high biomass and high cover lost more biomass to herbivores, as predicted by the Resource Concentration Hypothesis (Root 1973). Thus, showing that consumed biomass increases linearly with the amount of resources provided, while the percentage herbivory did not change in similar manners.

### Plant diversity effects on herbivory depend on resource availability and defense

Plant diversity effects on herbivory differed among species confirming other studies investigating the response of species-specific herbivory to increasing plant diversity (Koricheva et al. 2000; Scherber et al. 2006; Hahn et al. 2017). Effects ranged from positive to negative, caused by differences in biomass and the change in LDMC with plant diversity. Herbivory in plant species with high biomass increased with increasing plant diversity. Thus, either herbivores find their host plants easier and accumulate (Resource Concentration Hypothesis; Root 1973) or more resources support a higher herbivore abundance (More Individual Hypothesis; Srivastava and Lawton 1998; Borer et al. 2012). In both cases, herbivory would maintain or increase plant diversity, by decreasing the biomass of the most dominant species more efficiently than the biomass of subordinate species. However, the effect of resource availability (biomass) was surprisingly weak and relative abundance (cover) had no effect at all, which might be caused by the plants associated herbivores and differences in the average attractiveness to local herbivores (plant–herbivore interactions) and its impacts on associational effects (plant–plant interactions; Hambäck et al. 2014; Underwood et al. 2020). Alternatively, the difference between resource availability and relative abundance might indicate that supporting higher herbivore abundances with more plant biomass (More Individual Hypothesis) is more important than being apparent for herbivores with higher plant cover (Resource Concentration Hypothesis). Changes in LDMC with plant diversity explained additional variability in herbivory–plant diversity relationships of plant species. Specifically, plant species that increased their physical resistance became less damaged, while plant species that decreased their physical resistance became more damaged at higher plant diversity. This twofold pattern can be explained by plant species balancing costs of herbivory defense (e.g., adjusting defense traits) and growth (e.g., adjusting photosynthetic efficiency) and that impacts their palatability towards herbivores (Lind et al. 2013). In some cases, it could be beneficial for a plant to invest more into defense to increase herbivory resistance; in others, it may make sense to invest in growth and thereby improve competitive abilities. In addition, plant diversity effects on herbivory varied among years and seasons, which reflects fluctuations in herbivore populations, variability in plant performance, and differences in climate (Huntly 1991).

### Forbs and grasses differ in their herbivory

Forbs and grasses showed distinct patterns in both their average herbivory and their change in herbivory with plant diversity. Average herbivory was three times higher in forb species compared to grasses, most likely caused by higher palatability and lower physical resistance of forbs compared to grasses (Lavorel and Garnier 2002; Firn et al. 2019). Similar ratios were found by others (Turcotte et al. 2014; Fricke et al. 2022). Furthermore, plant traits explained differences among grasses, but not among forbs. In addition, we found different mechanisms explaining variability of plant diversity effects among forb species and among grasses. In detail, variability among forbs was best explained by physical resistance, matching the expectation that better-defended forbs should profit from increasing plant diversity by lower herbivory losses, because chances are higher that other plants of the same community are less defended and thus preferred by herbivores (Alm Bergvall et al. 2006; Underwood et al. 2014). In contrast, variability among grasses was best explained by resource apparency and might depend on the similarity of grass species, causing herbivores to forage in density-dependent manners rather than specializing (Massey et al. 2007; Hartley and DeGabriel 2016). Thus, in a plant community, herbivory could have strong implications for the relative abundance of forb species, while the effect of herbivory on the relative abundance of grasses might be weak.

### Summary and conclusions

We showed that the absolute herbivory differed between plant species and between and within functional groups, caused by variation in plant traits and apparency. Thus, herbivory might change plant community composition by targeting plants with specific properties. For example, we found that grasses that are well defended and not as palatable as forbs might benefit from herbivory. Furthermore, we showed that plant diversity effects on herbivory differed between plant species and within functional groups, posing different implications for grasslands dominated by either forbs or grasses. While the diversity of grass species might be maintained by herbivory, herbivory of forbs might cause less-defended species to decline and better-defended species to persist. Finally, the pronounced differences we found in plant–herbivore relationships among plant species suggest that community-level effects of herbivory depend on the plant species growing in that community rather than on plant diversity per se, explaining the mixed results of previous studies.

## Supplementary Information

Below is the link to the electronic supplementary material.

Supplementary file1 (DOCX 11577 KB)

## Data Availability

The data are deposited in jexis (jexis.idiv.de): doi.org/10.25829/G01W-ZW24, doi.org/10.25829/6F3Y-3F71

## Code availability

The code is deposited in jexis (jexis.idiv.de): doi.org/10.25829/G01W-ZW24.
