# Supplementary material for: Effects of plant diversity on species-specific herbivory: patterns and mechanisms
Source: Oecologia. 2023 Mar 24;201(4):1053–66. doi: 10.1007/s00442-023-05361-6 (PMC10113292; doi:10.1007/s00442-023-05361-6)
Supplement: Supplementary file 1 — Supplementary file1 (DOCX 11577 KB) [file 442_2023_5361_MOESM1_ESM.docx]

**Title:**

Effects of plant diversity on species-specific herbivory – Patterns and Mechanisms

**Authors:**

M. Bröcher^*, 1^, A. Ebeling^1^, L. Hertzog^2^, C. Roscher^3, 4^, W. Weisser^5^, S. T. Meyer^5^

^1^Institute of Ecology and Evolution, University of Jena, Jena, Germany

^2^Thünen Institute of Biodiversity, Braunschweig, Germany

^3^Department of Physiological Diversity, Helmholtz Centre for Environmental Research - UFZ, Leipzig, Germany

^4^German Centre for Integrative Biodiversity Research (iDiv) Halle-Jena-Leipzig, Leipzig, Germany

^5^Terrestrial Ecology Research Group, School of Life Sciences, Technical University of Munich, Freising, Germany

^*^Corresponding author: maximilian.broecher@uni-jena.de, Phone: 0049-3641-949426, Fax: 0049-3641-949402**Supplement Table 1:** Variance compounds extracted from linear mixed-effect models that used either percentage herbivory or consumed biomass as measure of herbivory. Variance explained and the corresponding standard deviations are given for each random term that entered the respective model.

|  |  | percentage herbivory | |  | consumed biomass | |  |
| --- | --- | --- | --- | --- | --- | --- | --- |
| model | random term | variance | σ |  | variance | σ |  |
| herbivory | plot | 0.091 | 0.302 |  | 0.075 | 0.273 |  |
| ~ biomass*FG | species | 1.125 | 1.061 |  | 0.988 | 0.994 |  |
|  | sampling time | 0.253 | 0.503 |  | 0.201 | 0.448 |  |
|  | residual | 3.459 | 1.860 |  | 3.346 | 1.829 |  |
| herbivory | plot | 0.092 | 0.303 |  | 0.068 | 0.262 |  |
| ~ cover*FG | species | 1.117 | 1.057 |  | 1.808 | 1.345 |  |
|  | sampling time | 0.256 | 0.506 |  | 0.341 | 0.584 |  |
|  | residual | 3.457 | 1.859 |  | 4.516 | 2.125 |  |
| herbivory | plot | 0.089 | 0.298 |  | 0.249 | 0.499 |  |
| ~ N-concentration*FG | species | 1.086 | 1.042 |  | 2.130 | 1.460 |  |
|  | sampling time | 0.254 | 0.504 |  | 0.344 | 0.587 |  |
|  | residual | 3.465 | 1.861 |  | 4.815 | 2.194 |  |
| herbivory | plot | 0.079 | 0.281 |  | 0.277 | 0.526 |  |
| ~ LDMC*FG | species | 1.072 | 1.035 |  | 2.222 | 1.491 |  |
|  | sampling time | 0.256 | 0.506 |  | 0.326 | 0.571 |  |
|  | residual | 3.463 | 1.861 |  | 4.794 | 2.190 |  |
| Pool A - herbivory | plot:block | 0.072 | 0.269 |  | 0.012 | 0.111 |  |
| ~ PSR*species*year*season | block | 0.034 | 0.185 |  | 0.033 | 0.182 |  |
|  | residual | 2.659 | 1.630 |  | 3.396 | 1.843 |  |
| Pool B - herbivory | plot:block | 0.050 | 0.224 |  | 0.068 | 0.260 |  |
| ~ PSR*species*year*season | block | 0.000 | 0.000 |  | 0.003 | 0.055 |  |
|  | residual | 2.361 | 1.537 |  | 2.992 | 1.730 |  |
| Pool C - herbivory | plot:block | 0.053 | 0.230 |  | 0.156 | 0.395 |  |
| ~ PSR*species*year*season | block | 0.034 | 0.185 |  | 0.041 | 0.202 |  |
|  | residual | 2.555 | 1.560 |  | 3.437 | 1.854 |  |
| PSR slope herbivory | sampling time | 0.020 | 0.143 |  | 0.000 | 0.000 |  |
| ~ biomass*FG | Residual | 0.877 | 0.936 |  | 1.304 | 1.142 |  |
| PSR slope herbivory | sampling time | 0.037 | 0.194 |  | 0.007 | 0.085 |  |
| ~ cover*FG | Residual | 0.882 | 0.939 |  | 1.321 | 1.149 |  |
| PSR slope herbivory | sampling time | 0.042 | 0.206 |  | 0.010 | 0.100 |  |
| ~ N-concentration*FG | Residual | 0.879 | 0.938 |  | 1.318 | 1.148 |  |
| PSR slope herbivory | sampling time | 0.035 | 0.186 |  | 0.002 | 0.043 |  |
| ~ LDMC*FG | Residual | 0.857 | 0.926 |  | 1.300 | 1.140 |  |

**Supplement Table 2:** Summary statistics for linear mixed-effects models testing the effect of biomass, cover, leaf N-concentration and LDMC and their interaction with functional group identity (FG) on consumed biomass (Figure S1) and the change in consumed biomass with plant species richness (PSR) (Figure S4). For each plant parameter we fitted a separate model. Asterisks indicate significance: *P < 0.05, **P < 0.01, ***P < 0.001.

|  |  |  |  |  |  |  |  |  |  |  |
| --- | --- | --- | --- | --- | --- | --- | --- | --- | --- | --- |
|  |  | Consumed biomass | | |  | PSR slope consumed biomass | | | |  |
| Explanatory variable |  | *F* value | | |  | *F* value | | | |  |
| biomass |  | *F*_1,2486_ | = | 1457.8*** |  |  | *F*_1,240_ | = | 2.8 |  |
| FG |  | *F*_1,20_ | = | 11.4** |  |  | *F*_1,240_ | = | 0.2 |  |
| FG:biomass |  | *F*_1,2999_ | = | 59.9** |  |  | *F*_1,240_ | = | 4.5* |  |
| cover |  | *F*_1,605_ | = | 282.4*** |  |  | *F*_1,238_ | = | 0.1 |  |
| FG |  | *F*_1,20_ | = | 6.6* |  |  | *F*_1,230_ | = | 0.7 |  |
| FG:cover |  | *F*_1,2095_ | = | 1.9 |  |  | *F*_1,240_ | = | 0.9 |  |
| N-concentration |  | *F*_1,38_ | = | 10.1** |  |  | *F*_1,233_ | = | 1.2 |  |
| FG |  | *F*_1,20_ | = | 5.2* |  |  | *F*_1,230_ | = | 0.0 |  |
| FG:N-concentration |  | *F*_1,2525_ | = | 0.1 |  |  | *F*_1,232_ | = | 2.0 |  |
| LDMC |  | *F*_1,23_ | = | 4.2 |  |  | *F*_1,231_ | = | 3.3 |  |
| FG |  | *F*_1,21_ | = | 6.1* |  |  | *F*_1,231_ | = | 1.1 |  |
| FG:LDMC |  | *F*_1,2450_ | = | 3.7 |  |  | *F*_1,240_ | = | 3.6 |  |
|  |  |  |  |  |  |  |  |  |  |  |

|  |  |  | |  |  |  |  |  |  |  |  |  |  |  | |  | | |
| --- | --- | --- | --- | --- | --- | --- | --- | --- | --- | --- | --- | --- | --- | --- | --- | --- | --- | --- |
|  |  |  | Consumed biomass | | | | | | | | | | | |  | | |  |
|  |  | Pool A | | | |  | Pool B | | |  | Pool C | | | |  | | |  |
| Explanatory variable |  | *F* value | | | |  | *F* value | | |  | *F* value | | | |  | |  |  |
| PSR |  | *F*_1,26_ | | = | 15.4*** |  | *F*_1,39_ | = | 32.2*** |  | *F*_1,42_ | = | | 23.2*** |  | |  |  |
| species |  | *F*_7,432_ | | = | 157.1*** |  | *F*_7,545_ | = | 79.0*** |  | *F*_7,493_ | = | | 76.2*** |  | |  |  |
| year |  | *F*_4,1039_ | | = | 10.7*** |  | *F*_4,1042_ | = | 25.1*** |  | *F*_4,884_ | = | | 5.2*** |  | |  |  |
| season |  | *F*_1,1040_ | | = | 27.0*** |  | *F*_1,1042_ | = | 28.4*** |  | *F*_1,885_ | = | | 0.4 |  | |  |  |
| PSR:species |  | *F*_7,440_ | | = | 4.2*** |  | *F*_7,422_ | = | 2.9** |  | *F*_7,351_ | = | | 2.3* |  | |  |  |
| PSR:year |  | *F*_4,1038_ | | = | 1.3 |  | *F*_4,1043_ | = | 0.8 |  | *F*_4,885_ | = | | 1.4 |  | |  |  |
| species:year |  | *F*_28,1037_ | | = | 5.4*** |  | *F*_28,1041_ | = | 4.7*** |  | *F*_28,884_ | = | | 5.3*** |  | |  |  |
| PSR:season |  | *F*_1,1042_ | | = | 2.6 |  | *F*_1,1045_ | = | 4.7* |  | *F*_1,884_ | = | | 0.5 |  | |  |  |
| species:season |  | *F*_7,1039_ | | = | 8.4*** |  | *F*_7,1041_ | = | 9.6*** |  | *F*_7,883_ | = | | 15.1*** |  | |  |  |
| year:season |  | *F*_4,1037_ | | = | 15.5*** |  | *F*_4,1041_ | = | 16.0*** |  | *F*_4,883_ | = | | 9.3*** |  | |  |  |
| PSR:species:year |  | *F*_28,1037_ | | = | 1.9** |  | *F*_28,1043_ | = | 0.9 |  | *F*_28,885_ | = | | 2.1*** |  | |  |  |
| PSR:species:season |  | *F*_7,1044_ | | = | 1.3 |  | *F*_7,1042_ | = | 0.5 |  | *F*_7,884_ | = | | 3.7*** |  | |  |  |
| PSR:year:season |  | *F*_4,1043_ | | = | 1.7 |  | *F*_4,1042_ | = | 0.2 |  | *F*_4,885_ | = | | 0.8 |  | |  |  |
| species:year:season |  | *F*_28,1038_ | | = | 3.8*** |  | *F*_28,1043_ | = | 2.3*** |  | *F*_28,886_ | = | | 2.7*** |  | |  |  |
| PSR:species:year:season |  | *F*_28,1039_ | | = | 1.4 |  | *F*_28,1044_ | = | 1.6* |  | *F*_28,889_ | = | | 1.3 |  | |  |  |
|  |  |  | |  |  |  |  |  |  |  |  |  |  |  | |  | | |

**Supplement Table 3:** Summary statistics for linear mixed-effects models testing the effect of plant species richness (PSR), species identity, year, season and all interactions on consumed biomass for the three different species pools separately. Asterisks indicate significance: *P < 0.05, **P < 0.01, ***P < 0.001.

**Supplement Table 4:** Summary statistics for linear models testing the effect of the change in consumed biomass with plant species richness (slope of consumed biomass and plant species richness (PSR)) on the change in biomass, cover, leaf N-concentration and LDMC with PSR (slope of biomass/cover/N-concentration/LDMC and PSR) and their interactions with functional group identity (FG). Asterisks indicate significance: *P < 0.05, **P < 0.01, ***P < 0.001.

|  |  |  |  |  |  | | |  |
| --- | --- | --- | --- | --- | --- | --- | --- | --- |
|  |  | PSR slope consumed biomass | | | |  |  |  |
| Explanatory variable |  | *F* value | | | |  |  |  |
| PSR slope biomass |  |  | *F*_1,20_ | = | 24.1*** | | |  |
| FG |  |  | *F*_1,20_ | = | 1.6 | | |  |
| FG:PSR slope biomass |  |  | *F*_1,20_ | = | 0.0 | | |  |
| PSR slope cover |  |  | *F*_1,20_ | = | 0.0 | | |  |
| FG |  |  | *F*_1,20_ | = | 0.1 | | |  |
| FG:PSR slope cover |  |  | *F*_1,20_ | = | 0.3 | | |  |
| PSR slope N-concentration |  |  | *F*_1,20_ | = | 0.4 | | |  |
| FG |  |  | *F*_1,20_ | = | 0.1 | | |  |
| FG:PSR slope N-concentration |  |  | *F*_1,20_ | = | 0.0 | | |  |
| PSR slope LDMC |  |  | *F*_1,20_ | = | 8.7** | | |  |
| FG |  |  | *F*_1,20_ | = | 0.1 | | |  |
| FG:PSR slope LDMC |  |  | *F*_1,20_ | = | 0.8 | | |  |
|  |  |  |  |  |  | | |  |





**Supplement Fig. 1** Effect of a) biomass, b) cover, c) N-concentration and d) LDMC on consumed biomass. Each point represents one species x pool combination. The species pool is indicated by the shape of the points (A=circle, B=triangle, C=square). Lines are predictions from the models and indicate significance (solid lines: P < 0.05; dashed lines: P > 0.05, Table S2). Response of herbivory for the average of both plant functional groups are shown in black, for forbs in blue, for grasses in red. Vertical and horizontal grey lines indicate the standard deviation per species. X-Axis of the upper left figure and all Y-Axes are logarithmic

**
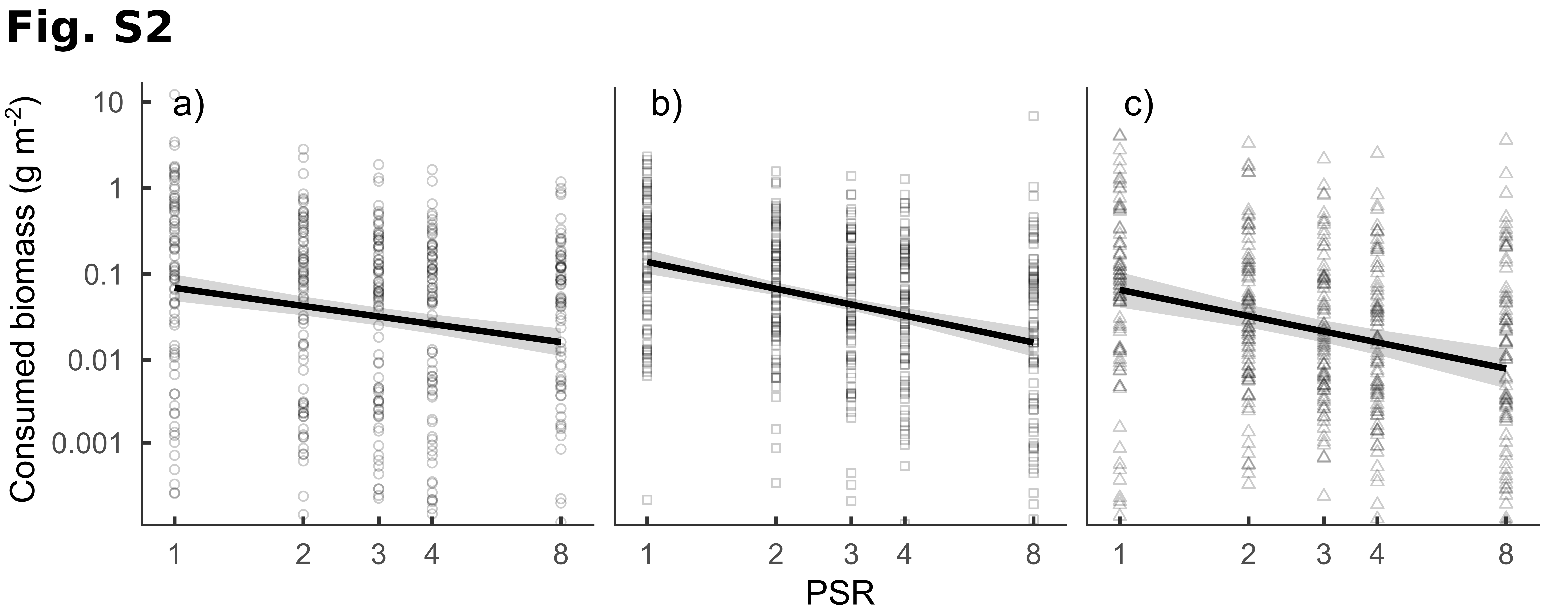
**

**Supplement Fig. 2** Effect of plant species richness on consumed biomass for three different species pools. Lines are predictions from the models and indicate significance (solid lines: P < 0.05; dashed lines: P > 0.05, Table S3). Bands indicate 95% confidence intervals. Axes are logarithmic

**
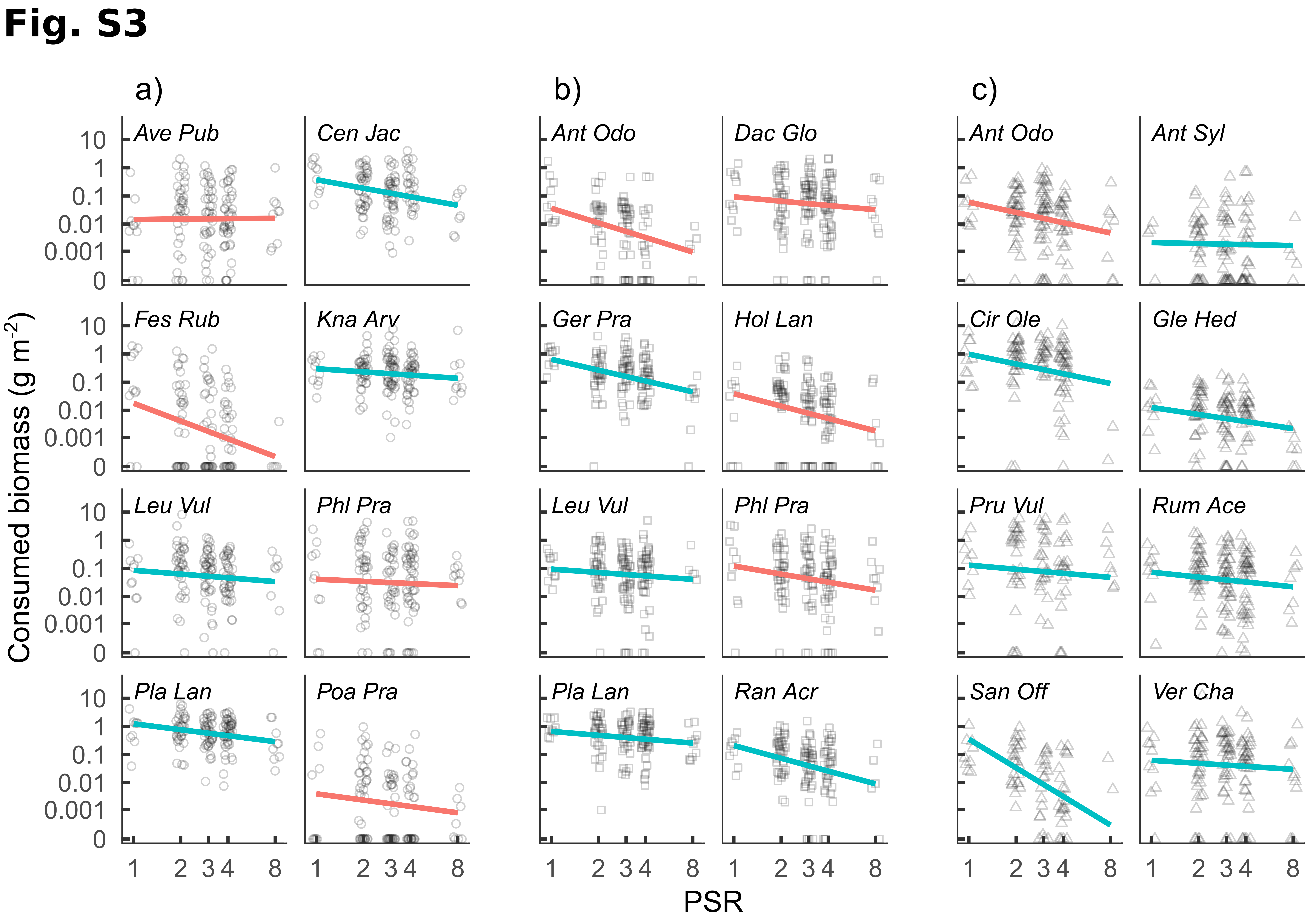
**

**Supplement Fig. 3** Effect of plant species richness on consumed biomass for all species x pool combinations. Lines are predictions from the models and the color of the lines indicate the functional group with forbs shown in blue and grasses in red. Axes are logarithmic

**
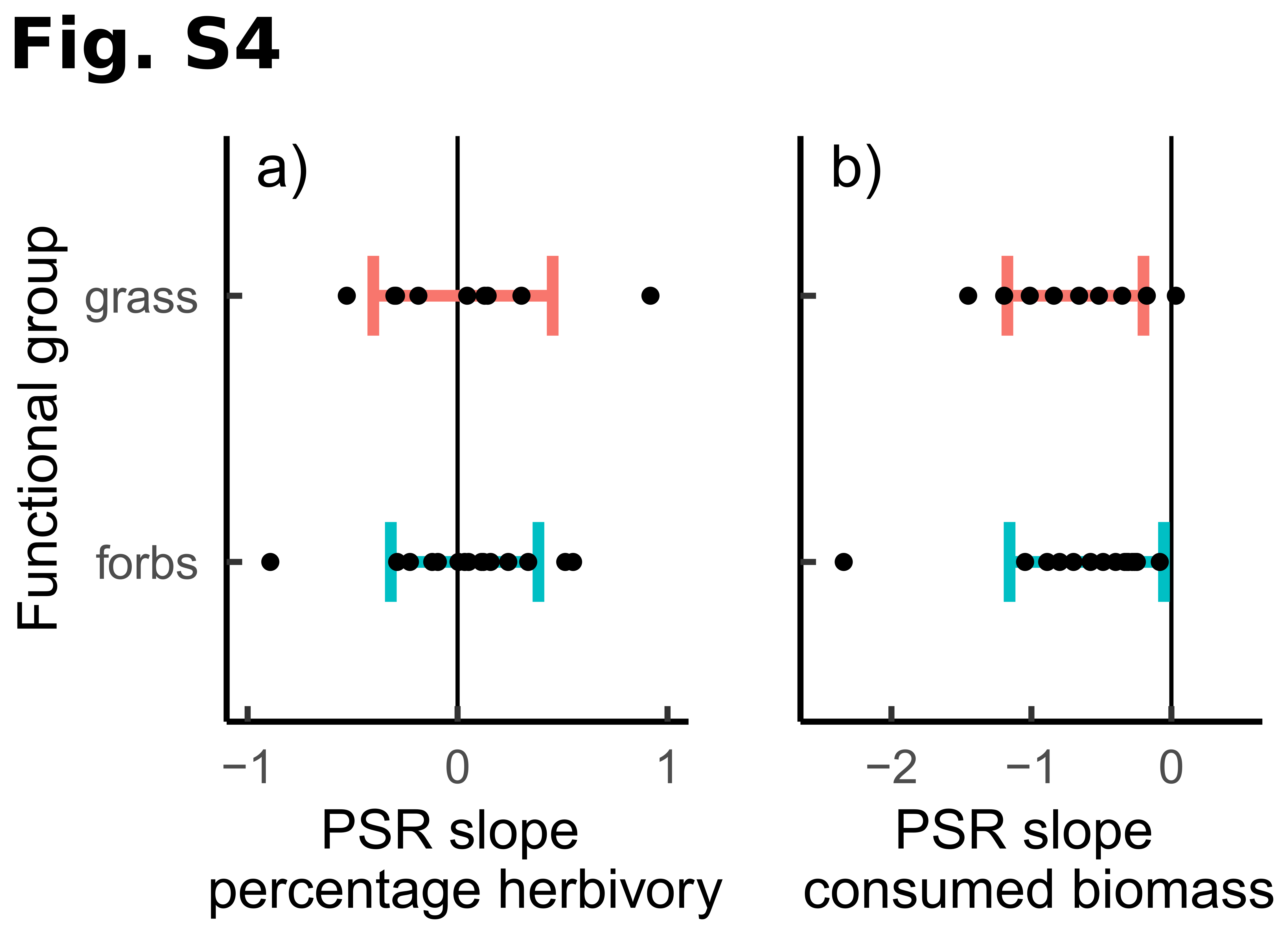
**

**Supplement Fig. 4** Average change in a) percentage herbivory and b) consumed biomass with plant species richness for forbs and grasses (slope of percentage herbivory or consumed biomass and plant species richness (PSR)). Forbs are shown in blue and grasses in red with their standard deviations, respectively

**

**

**Supplement Fig. 5** Effect of a) biomass, b) cover, c) leaf N-concentration and d) LDMC on the change in consumed biomass with plant species richness (PSR). Each point represents one species x pool combination. The species pool is indicated by the shape of the points (A=circle, B=triangle, C=square). Lines are predictions from the models and indicate significance (solid lines: P < 0.05; dashed lines: P > 0.05, Table S2). Response of herbivory for the average of both plant functional groups are shown in black, for forbs in blue, for grasses in red. Vertical and horizontal grey lines indicate the standard deviation per species. X-Axis of the upper left figure and all Y-Axes are logarithmic

**

**

**Supplement Fig. 6** Effect of species-specific change in a) biomass, b) cover, c) leaf N-concentration and d) LDMC with plant species richness (PSR) on the change in consumed biomass with PSR. Each point represents one species x pool combination. The species pool is indicated by the shape of the points (A=circle, B=triangle, C=square). Lines are predictions from the models and indicate significance (solid lines: P < 0.05; dashed lines: P > 0.05, Table S4). Response of herbivory for the average of both plant functional groups are shown in black, for forbs in blue, for grasses in red
